# Hepatic arterial chemotherapy infusion combined with tyrosine kinase inhibitors and PD-1 inhibitors for advanced hepatocellular carcinoma with high risk: a propensity score matching study

**DOI:** 10.1097/JS9.0000000000001940

**Published:** 2024-07-12

**Authors:** Mengxuan Zuo, Guanglei Zheng, Yuzhe Cao, Hailei Lu, Da Li, Chao An, Weijun Fan

**Affiliations:** a Department of Minimally Invasive Interventional Therapy, Sun Yat-sen University Cancer Center, Guangzhou; b State Key Laboratory of Oncology in South China, Guangzhou; c Guangdong Provincial Clinical Research Center for Cancer, Guangzhou People’s Republic of China

**Keywords:** advanced hepatocellular carcinoma, hepatic arterial infusion chemotherapy, high risk, programmed cell death protein-1 inhibitors, propensity score matching, triple therapy, tyrosine kinase inhibitors

## Abstract

**Objective:**

To ascertain the therapeutic efficacy and safety of FOLFOX (oxaliplatin, fluorouracil, and leucovorin)-based hepatic arterial infusion chemotherapy combined with tyrosine kinase inhibitors (TKI) and programmed cell death protein-1 inhibitors (PD-1 inhibitors) (triple therapy), as a first-line treatment in high-risk advanced hepatocellular carcinoma (aHCC with Vp4 portal vein invasion or/and tumor diameter ≥10 cm).

**Methods:**

This retrospective multicenter study included 466 high-risk aHCC patients treated with either triple therapy (*n*=245) or dual therapy (TKI and PD-1 inhibitors, *n*=221). The overall survival, progression-free survival, objective response rate, and safety were compared between the two groups. Propensity score matching was performed to reduce bias between the two groups.

**Results:**

After propensity score matching (1:1), 194 patients in each group were analyzed. The triple-therapy group showed a longer median overall survival (24.6 vs. 11.9 months; HR=0.43, *P*<0.001) and a longer median progression-free survival (10.0 vs. 7.7 months; HR=0.68, *P*=0.002) than the dual-therapy group. The survival rates at 6, 12, and 24 months were 94.2, 71.0, and 50.8% for triple therapy and 75.9, 49.9, and 26.8% for dual therapy. The objective response rate in the triple-therapy group was significantly higher (57.7 vs. 28.9%, *P*<0.001). In the triple-therapy group, more patients converted to non-high-risk (68.0 vs. 36.6%, *P*<0.001) and received salvage liver resection or ablation after downstaging conversion (16.5% vs. 9.2%, *P*=0.033). The grade 3/4 adverse events were 59.2 and 47.4% in the triple-therapy group and dual-therapy group, respectively (*P*=0.022).

**Conclusion:**

FOLFOX-based hepatic arterial infusion chemotherapy plus TKI and PD-1 inhibitors significantly improve survival prognosis compared with TKI plus PD-1 inhibitors. This is a potential first-line treatment for high-risk aHCC, with a relatively controlled safety profile.

## Introduction

Highlights

- The treatment options for high-risk hepatocellular carcinoma (HCC) patients (Vp4 portal vein invasion and/or tumor diameter ≥10 cm) were limited.
- Triple therapy significantly improves survival outcomes in high-risk advanced hepatocellular carcinoma (aHCC) patients with an acceptable safety profile compared with tyrosine kinase inhibitor (TKI) plus programmed cell death protein-1 inhibitors (PD-1 inhibitors).
- Conversion to non-high-risk and tumor downstaging were more common in triple therapy.

Primary liver cancer is the sixth most commonly diagnosed malignancy and the third leading cause of cancer death worldwide^1^. Most patients are found to have aHCC at the time of first diagnosis and have lost the chance of surgical resection, with a median survival of only 2.7–4.0 months^2^. Atezolizumab plus bevacizumab was recommended as first-line therapy for aHCC due to the high overall survival (OS) compared to sorafenib (19.2 vs. 13.4 months) in IMbrave 150^3,4^. Many other phase 3 randomized controlled trials (RCTs), such as LEAP-002, ORIENT-32, and CARES-310, also confirmed that the combination of TKI and immune checkpoint inhibitors were effective and safe^5–7^.

However, most RCTs focused on systemic treatment have excluded the patients with high risk, which is defined as Vp4 portal vein invasion and/or tumor occupancy of ≥50% of the liver, because of limited therapeutic response^5–7^. Although the high-risk population was enrolled in IMbrave 150, the median OS was only 7.6 months in the combination therapy group, which was much lower than that in the non-high-risk population^8^. There remains an unmet medical need to improve efficacy in patients with high-risk aHCC.

FOLFOX (fluorouracil, leucovorin, and oxaliplatin)-based hepatic arterial infusion chemotherapy (HAIC) was found to be an effective regional treatment for aHCC patients compared with sorafenib, with a median OS of 13.9 months^9^. Additionally, the combination of HAIC, TKI, and PD-1 inhibitors (one type of immune checkpoint inhibitors) (triple therapy) has shown promising efficacy and safety in several phase II trials, especially in patients with high tumor burden^10,11^. Unfortunately, triple therapy in high-risk aHCC with a large sample size has not been reported so far. Therefore, in this study, our objective is to compare the efficacy and safety of triple therapy and TKI plus PD-1 inhibitors (dual therapy) in first-line treatment for patients with high-risk aHCC, defined as Vp4 or/and tumor diameter ≥10 cm.

## Material and methods

### Study design and patients

The study was approved by the Ethics Committee and was performed by the Declaration of Helsinki. Given the retrospective nature of the study, informed consent was waived. The strengthening of the reporting of cohort, cross-sectional, and case–control studies in surgery (STROCSS, Supplemental Digital Content 1, http://links.lww.com/JS9/D100) guideline was followed, and the completed checklist was provided^12^. In addition, this study had been registered with the unique identifying number.

The retrospective study of multiple centers included high-risk aHCC patients treated with triple therapy (HAIC plus TKI and PD-1 inhibitors) or dual therapy (TKI plus PD-1 inhibitors) from October 2014 to April 2022. The inclusion criteria were as follows: patients were diagnosed pathologically or clinically according to the European Association for the Study of Liver criteria^13^; Barcelona Clinic Liver Cancer stage C patients with high risk (Vp4 or/and tumor diameter ≥10 cm); patients received triple therapy or dual therapy during the same period; aged 18–80 years; Eastern Cooperative Oncology Group performance status of 0 or 1; preserved liver function as Child–Pugh A or B class; and at least one measurable intrahepatic lesion based on modified Response Evaluation Criteria in Solid Tumors (mRECIST) criteria^14^. The exclusion criteria were as follows: history of another malignant tumor; incomplete pretreatment computed tomography (CT)/MRI image or clinical laboratory data; and incomplete follow-up data.

The multidisciplinary team recommended treatment options based on Barcelona Clinic Liver Cancer or China National Liver Cancer guidelines, individual conditions, and economic situation. Patients and their family members made the final decision together.

### Treatment protocol

The patients received four to six cycles of HAIC performed by interventional radiologists with at least 5 years of experience, as described in previous studies^10,15^. Patients repeatedly received HAIC with the FOLFOX regimen every 21-day cycle (oxaliplatin 85 mg/m^2^ infusion for 2 h; leucovorin 400 mg/m^2^ for 2–3 h; and fluorouracil 1250/2500 mg/m^2^ for 23/46 h). If the patients exhibited poor general condition (Eastern Cooperative Oncology Group >2), impaired liver function (Child–Pugh C), disease progression, or intolerable adverse events, HAIC would be discontinued. TKI and PD-1 inhibitors were received within 3 days after the first cycle of HAIC.

All patients received a standard dose of TKI orally every day. The type of TKI in this study includes lenvatinib (12 mg/day for body weight 60 kg or 8 mg/day for body weight <60 kg orally once daily), sorafenib (400 mg twice a day), apatinib (250 mg orally once daily), and donafenib (200 mg twice a day). PD-1 inhibitors, including camrelizumab, sintilimab, and tislelizumab, were administered intravenously at 200 mg every 3 weeks. Another PD-1 inhibitor, toripalimab, was administered intravenously at 240 mg every 3 weeks. Dose reduction or temporary interruption was allowed for TKI if patients experienced treatment-related adverse events, for example, malignant hypertension or severe hand–foot syndrome. For PD-1 inhibitors, dose reduction was not allowed, but temporary interruption was allowed because of adverse events. The TKI and PD-1 inhibitors were continued until disease progression, death, unacceptable toxicity occurred, patient demand, or loss of follow-up.

If the tumor goes downstage or converts from high-risk to non-high-risk, patients will be evaluated for subsequent therapy by multidisciplinary team. Salvage liver resection or ablation was recommended and performed by surgeons with 15 years of experience in resection or ablation. Patients made the final decision according to individual conditions.

### Follow-up

All patients received standardized assessments every 3–6 weeks. Comprehensive evaluations were performed at each follow-up to assess tumor response, including dynamic contrast-enhanced abdominal MRI/CT, dynamic contrast-enhanced chest CT, and laboratory tests. Laboratory tests included serum alpha-fetoprotein, des-γ-carboxy prothrombin, alanine transferase, bilirubin, albumin, prothrombin time, and platelet count. Second-line treatment after disease progression was performed according to HCC guidelines.

### Assessment of outcomes

Tumor responses were independently evaluated by two radiologists with 5 years of experience based on mRECIST, and senior radiologists made the final decision in the event of disagreement. For survival outcomes, the median OS (defined as the time from the first treatment to death for any reason) and progression-free survival (PFS, defined as the time from the first treatment to the first clinical progression or death) were the primary outcome and the secondary outcome in this study, respectively. Another secondary outcome was the tumor response, which was classified as complete response (CR), partial response (PR), stable disease, and progressive disease. Objective response rate (ORR) was defined as the proportion of patients with CR or PR. Disease control rate was defined as ORR plus stable disease rate. Adverse events were evaluated by Common Terminology Criteria for Adverse Events (CTCAE), version 5.0.

### Statistical analysis

Propensity score matching (PSM) was performed to balance the confounders of baseline characteristics of the patients and to reduce bias between the triple- and dual-therapy groups. Factors in PSM included age, sex, hepatitis B infection, Child–Pugh class, alpha-fetoprotein, tumor size, tumor number, Vp4, and distant metastases. The match ratio is 1:1 and the caliper scores are 0.02.

Continuous variables with mean±SD or median with interquartile range (IQR) were compared using a two-sided independent *t* test or Wilcoxon rank-sum test. Categorical variables were compared using the *χ* ^2^ test. OS and PFS were analyzed and compared using the Kaplan–Meier curves and the logarithmic rank test. Univariate and multivariate Cox regression was applied to identify independent risk factors for OS and PFS using the Cox proportional hazards model.

Two-sided *P* values <0.05 were considered statistically significant. All statistical and PSM analyzes were performed using SPSS (version 25.0) or R (version 4.3.0).

## Results

### Patient characteristics

**Figure 1:**
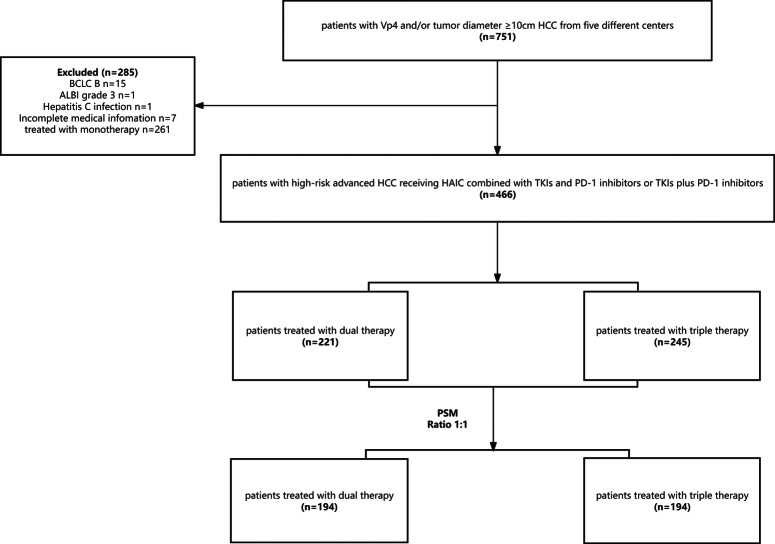
Flowchart of high-risk advanced HCC patients who received triple or dual therapy. HAIC, hepatic arterial infusion chemotherapy; HCC, hepatocellular carcinoma; PD-1, programmed cell death protein-1; TKI, tyrosine kinase inhibitor.

The patient-enrolled flow chart is illustrated in Figure 1. Between October 2014 and April 2022, 751 patients with high-risk aHCC were diagnosed in five medical centers. Among them, 245 patients received triple therapy, and 221 received dual therapy. The baseline characteristics of the patients are presented in Table 1. Overall, 402 (402/466, 86.3%) patients were male individuals, 217 (217/466, 46.6%) patients >50 years old, and 424 (424/466, 91.0%) patients had hepatitis B virus. There were 155 (155/466, 33.3%) patients with portal vein invasion of the Vp4 and 396 (396/466, 85%) patients with a maximum tumor diameter ≥10 cm, with a median tumor size of 12.1 cm. The triple-therapy group had a significantly high rate of distant metastasis (54.7 vs. 44.3%, *P*=0.032). After PSM in a 1:1 ratio, the total number of matched patients was 388, and baseline characteristics were further balanced between the matched cohorts (*P*>0.05 for all characteristics). In the PSM data, the median follow-up time of the triple-therapy and dual-therapy groups was 12.7 months (IQR, 9.0–20.2) and 9.6 months (IQR, 6.0–13.1), respectively. The median HAIC cycle was 5 in the triple-therapy group.

**Table 1:** Baseline characteristics of patients.

	Before matching				After matching			
Characteristic	Dual therapy (*N*=221)	Triple therapy (*N*=245)	Total (*N*=466)	*P*	Dual therapy (*N*=194)	Triple therapy (*N*=194)	Total (*N*=388)	*P*
Age (years)								
≤50	116 (52.5%)	133 (54.3%)	249 (53.4%)	0.768	98 (50.5%)	92 (47.4%)	190 (49.0%)	0.612
>50	105 (47.5%)	112 (45.7%)	217 (46.6%)		96 (49.5%)	102 (52.6%)	198 (51.0%)	
Sex								
Female	39 (17.6%)	25 (10.2%)	64 (13.7%)	0.028	27 (13.9%)	21 (10.8%)	48 (12.4%)	0.441
Male	182 (82.4%)	220 (89.8%)	402 (86.3%)		167 (86.1%)	173 (89.2%)	340 (87.6%)	
Hepatitis B infection								
No	24 (10.9%)	18 (7.3%)	42 (9.0%)	0.246	15 (7.7%)	17 (8.8%)	32 (8.2%)	0.854
Yes	197 (89.1%)	227 (92.7%)	424 (91.0%)		179 (92.3%)	177 (91.2%)	356 (91.8%)	
ALBI grade								
1	119 (53.8%)	115 (46.9%)	234 (50.2%)	0.163	106 (54.6%)	95 (49.0%)	201 (51.8%)	0.31
2	102 (46.2%)	130 (53.1%)	232 (49.8%)		88 (45.4%)	99 (51.0%)	187 (48.2%)	
AFP (ng/ml)								
≤400	87 (39.4%)	84 (34.3%)	171 (36.7%)	0.298	71 (36.6%)	79 (40.7%)	150 (38.7%)	0.466
>400	134 (60.6%)	161 (65.7%)	295 (63.3%)		123 (63.4%)	115 (59.3%)	238 (61.3%)	
Child–Pugh class								
A	199 (90.0%)	222 (90.6%)	421 (90.3%)	0.96	173 (89.2%)	174 (89.7%)	347 (89.4%)	1
B	22 (10.0%)	23 (9.4%)	45 (9.7%)		21 (10.8%)	20 (10.3%)	41 (10.6%)	
Tumor size, cm								
Median (IQR)	11.8 (10.0 to 15.1)	12.4 (10.7 to 14.8)	12.1 (10.3 to 14.9)	0.396	11.8 (10.0 to 15.5)	12.0 (10.3 to 14.0)	12.0 (10.1 to 14.8)	0.815
<10	31 (14.0%)	39 (15.9%)	70 (15.0%)	0.659	30 (15.5%)	33 (17.0%)	63 (16.2%)	0.783
≥10	190 (86.0%)	206 (84.1%)	396 (85.0%)		164 (84.5%)	161 (83.0%)	325 (83.8%)	
Tumor number								
≤3	95 (43.0%)	85 (34.7%)	180 (38.6%)	0.082	78 (40.2%)	82 (42.3%)	160 (41.2%)	0.757
>3	126 (57.0%)	160 (65.3%)	286 (61.4%)		116 (59.8%)	112 (57.7%)	228 (58.8%)	
Vp4								
No	157 (71.0%)	154 (62.9%)	311 (66.7%)	0.076	131 (67.5%)	119 (61.3%)	250 (64.4%)	0.243
Yes	64 (29.0%)	91 (37.1%)	155 (33.3%)		63 (32.5%)	75 (38.7%)	138 (35.6%)	
Vp4 and tumor size ≥ 10 cm								
No	187 (84.6%)	193 (78.8%)	380 (81.5%)	0.133	160 (82.5%)	152 (78.4%)	312 (80.4%)	0.371
Yes	34 (15.4%)	52 (21.2%)	86 (18.5%)		34 (17.5%)	42 (21.6%)	76 (19.6%)	
Distant metastasis								
No	123 (55.7%)	111 (45.3%)	234 (50.2%)	0.032	105 (54.1%)	106 (54.6%)	211 (54.4%)	1
Yes	98 (44.3%)	134 (54.7%)	232 (49.8%)		89 (45.9%)	88 (45.4%)	177 (45.6%)	

AFP, alpha-fetoprotein; IQR, interquartile range.

*P* values were calculated using a two-sided *χ* ^2^ test and Wilcoxon rank-sum test.

### Efficacy

**Figure 2:**
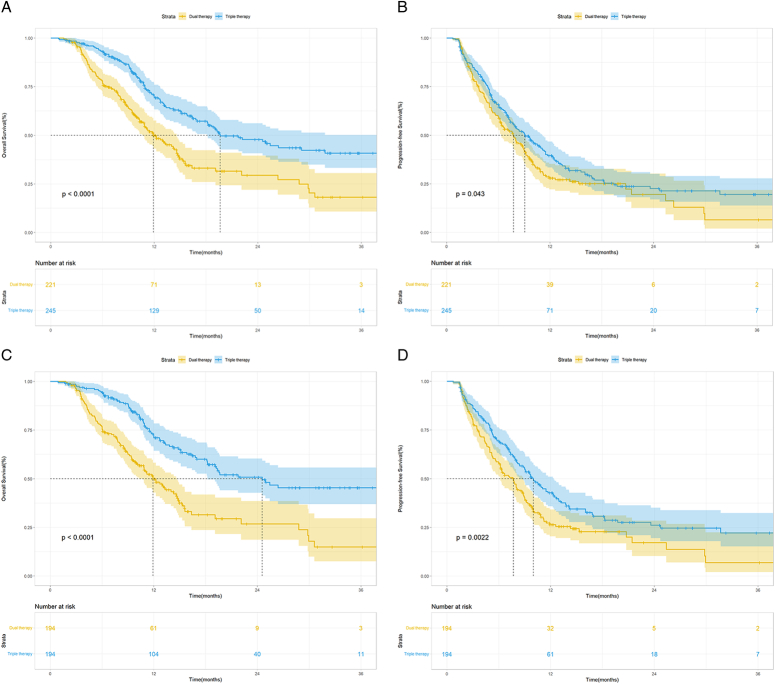
Kaplan–Meier survival curves of overall survival and progression-free survival in patients accepted dual therapy versus triple therapy before (A, B) and after (C, D) propensity score matching.

Triple therapy showed significantly higher median OS, PFS, and ORR than dual therapy (OS: 19.6 months [95% CI, 14.4–24.8] vs. 11.9 months [95% CI, 9.9–13.9], HR=0.50, *P*<0.001; PFS: 9.0 months [95% CI, 7.4–10.8] vs. 7.8 months [95% CI, 6.3–9.2], HR=0.80, *P*=0.044; ORR: 53.9% vs. 29.0%, *P*=0.002; Fig. 2A, B and Table 2). After 1:1 PSM, the median OS for the triple-therapy group and the dual-therapy group was 24.6 months (95% CI, 13.0–36.1) and 11.9 months (95% CI, 10.3–13.5), respectively (*P*<0.001; Fig. 2C). The median PFS was 10 months (95% CI, 8.5–11.5) and 7.7 months (95% CI, 6.4–9.0), respectively, for triple therapy and dual therapy (*P*=0.002). Survival rates at 6, 12, and 24 months were respectively 94.2, 71.0, and 50.8% for triple therapy and 75.9, 49.9, and 26.8% for dual therapy. Based on the mRECIST criteria, patients in the triple-therapy group showed a higher ORR than those in the dual-therapy group (57.7 vs. 28.9%, *P*<0.001; Table 2). In particular, 68.0% (132/194) patients turned non-high-risk after triple-therapy, while only 36.6% (71/194) patients reached non-high-risk in the dual-therapy group (*P*<0.001; Table 2). The triple-therapy group also had more patients (32/194, 16.5%) who received salvage liver resection or ablation after downstaging conversion than the dual-therapy group (18/194, 9.2%) (*P*=0.033; Table 2).

**Table 2:** Tumor response evaluated by modified Response Evaluation Criteria in Solid Tumors, conversion to non-high-risk rate and radical treatment (liver resection or ablation) rete.

	Before PSM				After PSM			
	Dual therapy (*N*=221)	Triple therapy (*N*=245)	Total (*N*=466)	*P*	Dual therapy (*N*=194)	Triple therapy (*N*=194)	Total (*N*=388)	*P*
CR	4 (1.8%)	5 (2%)	9 (1.9%)	1	2 (1%)	5 (2.6%)	7 (1.8%)	0.446
PR	60 (27.1%)	127 (51.8%)	187 (40.1%)	<0.001	54 (27.8%)	107 (55.2%)	161 (41.5%)	<0.001
SD	102 (46.2%)	82 (33.5%)	184 (39.5%)	0.007	87 (44.8%)	60 (30.9%)	147 (37.9%)	0.007
PD	55 (24.9%)	31 (12.7%)	86 (18.5%)	0.001	51 (26.3%)	22 (11.3%)	73 (18.8%)	<0.001
ORR	64 (29.0%)	132 (53.9%)	196 (42.1%)	<0.001	56 (28.9%)	112 (57.7%)	168 (43.3%)	<0.001
DCR	166 (75.1%)	214 (87.3%)	380 (81.5%)	0.001	143 (73.7%)	172 (88.7%)	315 (81.2%)	<0.001
Conversion	85 (38.5%)	149 (60.8%)	234 (50.2%)	<0.001	71 (36.6%)	132 (68.0%)	203 (52.3%)	＜0.001
Radical treatment	27(12.2%)	46(18.8%)	73 (15.7%)	0.014	18(9.2%)	32(16.5%)	50 (12.9%)	0.033

CR, complete response; DCR, disease control rate; ORR, overall response rate; PD, progressive disease; PR, partial response; PSM, propensity score matching; SD, stable disease.

*P* values were calculated using a two-sided *χ* ^2^ test.

**Figure 3:**
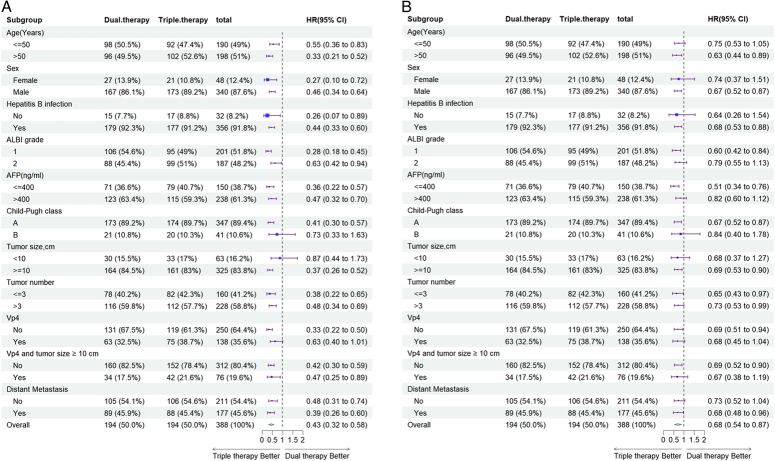
Forest plots of overall survival (A) and progression-free survival (B) in different subgroups after propensity score matching.CI, confidence interval; HR, hazard ratio.

Univariate and multivariate Cox regression analyses of PFS and OS after PSM are listed in Table 3. Multivariate analysis showed that triple therapy was significantly associated with longer OS and PFS than dual therapy (OS: HR=0.46; 95% CI, 0.34–0.62; *P*<0.001; for PFS, HR=0.71; 95% CI, 0.55–0.91; *P*=0.006; Table 3). The tumor number was another independent survival prognostic factor, while distant metastasis and tumor number were other independent progression prognostic factors. The results of the multivariate analysis were similar to those before the PSM matching (Supplementary Table 1, Supplemental Digital Content 2, http://links.lww.com/JS9/D101). The results of the subgroup analysis consistently demonstrated that triple therapy effectively had lower HR values in different subgroups characterized by tumor number >3, tumor size ≥10 cm, and distant metastasis (Fig. 3).

**Table 3:** Univariate and multivariate Cox regression analyses of the prognostic factors for overall survival and progression-free survival after propensity score matching.

	OS				PFS			
	Univariate cox regression		Multivariate cox regression		Univariate cox regression		Multivariate cox regression	
Variables	HR (95% CI)	*P*	HR (95% CI)	*P*	HR (95% CI)	*P*	HR (95% CI)	*P*
AFP, ng/ml (≤400 versus ＞400)	0.92 (0.69–1.24)	0.597			1.04 (0.81–1.34)	0.732		
Age, year (≤50 versus >50)	0.90 (0.67–1.20)	0.463			0.87 (0.68–1.10)	0.248		
ALBI (1 vs. 2)	1.23 (0.92–1.63)	0.166			0.96 (0.76–1.23)	0.755		
Child–Pugh class (A vs. B)	1.74 (1.16–2.62)	0.008	1.47 (0.98–2.23)	0.066	1.13 (0.77–1.66)	0.523		
Distant metastasis (absent vs. present)	1.22 (0.91–1.62)	0.181			1.32 (1.03–1.68)	0.027	1.26 (0.99–1.61)	0.05
Hepatitis B infection (absent vs. present)	1.18 (0.66–2.13)	0.573			1.08 (0.68–1.70)	0.755		
Sex (female vs. male)	0.87 (0.56–1.35)	0.533			0.87 (0.60–1.25)	0.439		
Tumor number (≤3 vs. ＞3)	1.71 (1.26–2.33)	0.001	1.60 (1.17–2.17)	0.003	1.46 (1.14–1.88)	0.003	1.40 (1.09–1.81)	0.008
Tumor size, cm (＜10 vs. ＞=10)	1.01 (0.69–1.47)	0.952			1.37 (0.98–1.92)	0.067	1.21 (0.80–1.85)	0.367
Vp4 (absent vs. present)	1.05 (0.78–1.41)	0.759			0.80 (0.62–1.03)	0.085	0.90 (0.65–1.25)	0.534
Vp4 and tumor size ≥10 cm (absent vs. present)	1.11 (0.78–1.58)	0.57			0.96 (0.71–1.32)	0.821		
Treatment (dural therapy vs. triple therapy)	0.43 (0.32–0.58)	<0.001	0.46 (0.34–0.62)	<0.001	0.68 (0.53–0.87)	0.002	0.71 (0.55–0.91)	0.006

AFP, alpha-fetoprotein; CI, confidence interval; HR, hazard radio; PFS, progression-free survival.

Any factors that were statistically significant at *P* value <10% in the univariate analysis were candidates for entry into a multivariable Cox analysis.

In the triple-therapy group, patients with better liver function, smaller tumor diameter, fewer distant metastasis, and more HAIC cycles exhibited better tumor response (CR and PR) after therapy (*P*<0.05; Supplementary Table 4, Supplemental Digital Content 2, http://links.lww.com/JS9/D101). Multivariate Cox regression analysis showed that HAIC cycle was an independent risk factor for OS (HR=0.75; 95% CI=0.66–0.84, *P*<0.001; Supplementary Table 5, Supplemental Digital Content 2, http://links.lww.com/JS9/D101) and PFS (HR=0.87; 95% CI=0.80–0.95, *P*=0.003; Supplementary Table 5, Supplemental Digital Content 2, http://links.lww.com/JS9/D101). Furthermore, patients with higher HAIC cycles (six or more cycles) significantly achieved better survival prognosis compared to those with lower HAIC cycles (less than six cycles) in the triple-therapy group (median OS: 40.6 months [95% CI, 19.6–not reached] vs. 15.1 months [95% CI, 12.0–25.4], *P*<0.001; median PFS: 10.5 months [95% CI, 8.2–13.4] vs. 7.7 months [95% CI, 5.5–11.2], *P*=0.078; Supplementary Figure 1, Supplemental Digital Content 2, http://links.lww.com/JS9/D101).

### Adverse events and safety

Of the 194 patients in each therapy group, 190 (190/194, 97.9%) patients in the triple-therapy group and 186 (186/194, 95.9%) patients in the dual-therapy group experienced treatment-related adverse events. High-frequency-grade adverse events after receiving triple therapy and dual therapy were elevated aspartate aminotransferase (314/388, 81.0%), elevated alanine aminotransferase (285/388, 73.5%), and hypertension (238/388, 61.3%). Grade 3/4 adverse events were recorded in 115 (115/194, 59.2%) of the triple-therapy group and 92 (92/194, 47.4%) of the dual-therapy group (*P*=0.022). The most common grade 3/4 adverse events were hypertension (107/388, 27.6%), thrombocytopenia (78/388, 20.1%), and neutropenia (73/388, 18.8%). All of these AEs were manageable, and treatment-related deaths did not occur in this study. Detailed adverse event profiles were summarized in Table 4 (Supplementary Tables 2 and 3, Supplemental Digital Content 2, http://links.lww.com/JS9/D101).

**Table 4:** Treatment-related adverse events.

	Before PSM				After PSM			
Adverse event	Dual therapy (*N*=221)	Triple therapy (*N*=245)	Total (*N*=466)	*P*	Dual therapy (*N*=194)	Triple therapy (*N*=194)	Total (*N*=388)	*P*
Grade 1–2	139 (62.9%)	198 (80.8%)	337 (72.3%)	<0.001	121 (62.4%)	154 (79.4%)	275 (70.9%)	<0.001
Grade 3–4	109 (49.3%)	150 (61.2%)	259 (55.6%)	0.013	92 (47.4%)	115 (59.2%)	207 (53.4%)	0.022
Any grade	212 (95.9%)	241 (98.4%)	453 (97.2%)	0.188	186 (95.9%)	190 (97.9%)	376 (96.9%)	0.379

PSM, propensity score matching.

*P* values were calculated using a two-sided *χ* ^2^ test.

## Discussion

The multicenter retrospective study showed that triple therapy of HAIC, combined with TKI and PD-1 inhibitors, significantly improved long-term OS and PFS in aHCC patients with high risk compared to dual therapy of TKI plus PD-1 inhibitors. Triple therapy exhibited a higher ORR with more patient down-risk. Multivariate Cox regression demonstrated that triple therapy was an independent positive prognostic indicator for long-term survival in patients. The subgroup analysis also showed consistent survival benefits among the groups. Triple therapy was found to have a significantly higher incidence of adverse events, but most of them were manageable.

The prognosis of aHCC patients with high risk remains suboptimal in the treatment of systemic therapy; therefore, the majority of studies related to systemic treatment have excluded those patients^16–18^. However, in the real world, many patients are at high risk once diagnosed, especially in China, where the median tumor diameter of HCC patients is 6.7 cm^19^. Previously, several studies focused on a combination of regional and systemic therapy have enrolled some patients with high risk^11,20,21^. However, the population included in these studies is heterogeneous, and the proportion of high-risk subgroups is relatively low. Our study is the first to evaluate the efficacy of HAIC plus TKI and PD-1 inhibitors in high-risk aHCC with a large sample size, and we also compared it with the first-line treatment recommended by the HCC guidelines. Furthermore, the reason for choosing HAIC over transcatheter arterial chemoembolization in this study was that transcatheter arterial chemoembolization had limited efficacy or is the relative contraindication in patients with portal vein tumor thrombosis of Vp4 or / and high tumor burden^2,22,23^.

The main cause of death in aHCC is intrahepatic lesions, which directly determine the patient’s survival time^24,25^. FOLFOX-HAIC can be an effective means to control intrahepatic lesions, which could induce tumor necrosis and release tumor antigens, and it has been proved that FOLFOX-HAIC is beneficial for prognosis in aHCC, compared to sorafenib^9,15^. In addition, the application of antiangiogenic agents induces vascular normalization and reduces tumor angiogenesis, which could promote immune cell infiltration^26^. All of these could convert the ‘cold tumor’ into a ‘hot tumor,’ and the tumor microenvironment is sensitive to immune therapy^27–29^. Triple therapy could contribute to synergistic benefits and better patient outcomes^10,30^.

In this research, patients receiving triple therapy demonstrated a median OS and PFS of 24.6 and 10.0 months, outperforming the result reported in the study of target therapy combined with immune therapy, HAIC, or HAIC combined with target therapy/immunotherapy. In the IMbrave 150 study, updated data revealed that the median OS in high-risk patients who received atezolizumab plus bevacizumab was only 7.6 months, compared to 5.5 months in high-risk patients who received sorafenib, which is poorer than in the normal population^8^. Triple therapy showed higher OS and PFS than in this study. Compared to HAIC monotherapy and HAIC plus sorafenib, median OS and PFS in patients with high-risk HCC ranged from 9.47 to 13.6 months and 6.8 to 7.7 months, all of which were lower than the results observed in our study^20,21^. A small-sample prospective study (Le-To-HAIC) investigating triple therapy in high-risk patients has reported a median OS of 17.9 months and a median PFS of 10.4 months^11^. In comparison, our study had a larger sample size and less heterogeneity, providing more reliable statistical evidence for the efficacy of triple therapy in high-risk patients.

Multivariate analysis identified triple therapy as an independent positive prognostic factor for both OS and PFS, highlighting the favorable impact of triple therapy on patient outcomes. The stratified analysis revealed that triple therapy improved survival time in most subgroups. We can see that most patients (132/194, 68%) converted to non-high-risk or downstage after triple therapy. Several studies have revealed that a combination of regional and systemic therapy could promote the rate of salvage resection after downstaging^31,32^. In our study, more patients (32/194, 16.5%) in the triple-therapy group received liver resection or ablation after downstaging. In addition, it is interesting that triple therapy is also more effective than dual therapy in patients with distant metastases. HAIC, as a regional therapy, is usually ineffective for extrahepatic metastases, so the possible explanation is that the synergistic effect of HAIC makes the lesion sensitive to TKI and PD-1 inhibitors in patients with extrahepatic metastases.

Based on the prognosis analysis of the triple-therapy group, it was revealed that HAIC cycle≥ 6 is strongly associated with positive outcomes. Similar to clinical experience and most previous studies^10,33^, it was recommended that six cycles of HAIC be administered, which could reach maximum efficacy without causing intolerable adverse reactions. In our study, the number of HAIC cycle is affected by various factors, such as the tumor progression, severe adverse events, patient compliance, and economic conditions. So more prospective studies are needed to explore the optimal HAIC cycles in combination therapy with HCC.

Grade ≥ 3 adverse events occurred in approximately 59.2 and 47.4% of the triple-therapy group and dual-therapy group, respectively. Although patients treated with TKI, PD-1 inhibitors, plus HAIC had significantly elevated frequencies of grades 3–4 adverse events, all patients can tolerate the full course of treatment. In the triple-therapy group, the most common grades 3–4 adverse events were neutropenia (30.4%), abdominal pain (28.4%), thrombocytopenia (25.3%), and increased alanine aminotransferase and aspartate aminotransferase (22.2 and 23.2% for each), which were consistent with those that occurred in a previous study^10^. Chemotherapeutic agents caused these treatment-related adverse events and were manageable by dosage modification or temporary interruption. The TKI-related and immune-related treatment-related adverse events, such as hand–foot syndrome and hypothyroidism, occurred in 42.8 and 9.8% of the patients, which were comparable with the dual-therapy group. The combination of these three treatments was clinically feasible and safe, with no treatment-related death occurring.

This study has several limitations that should be acknowledged: first, as a retrospective study rather than a prospective study, the presence of confounding biases is inevitable. To balance confounders, we employed methods such as PSM, subgroup analysis, and multivariate regression to ensure the persuasiveness of our data. Second, the combinations of TKI plus PD-1 inhibitors in this study were diverse, and no specific combination was used. Third, there are no follow-up biomarkers to monitor the efficacy of triple therapy.

In conclusion, these findings suggest that triple therapy is a promising and potential first-line treatment option with an acceptable safety profile for high-risk HCC patients, and to strongly identify the study points, more prospective large-scale RCTs need to be conducted in the future.

## Ethical approval

The study was approved by the Ethics Committee of the Sun Yat-sen University Cancer Center (approved ethics number: B2023-358-01).

## Consent

Given the retrospective nature of the study, the Ethics Committee of the Sun Yat-sen University Cancer Center allowed informed consent to be waived.

## Source of funding

There were no funding sources for this study.

## Author contribution

W.F. and C.A. conceived and designed the experiments, reviewed the manuscript, and approved the final draft. M.Z., G.Z., and Y.C. analyzed the data, authored the original manuscript, and approved the final draft. H.L. and D.L. collected and analyzed the data and approved the final draft.

## Conflicts of interest disclosure

The authors declare no conflicts of interest.

## Research registration unique identifying number (UIN)

The study had been registered in the Chinese Clinical Trial Registry (ChiCTR), with the Unique Identifying Number: ChiCTR2300077552 (https://www.chictr.org.cn/).

## Guarantor

Weijun Fan, Chao An, Mengxuan Zuo, Guanglei Zheng, and Yuzhe Cao.

## Data availability statement

The data that support the findings of this study are available from the corresponding author upon reasonable request.

## Provenance and peer review

The paper was not invited.

## Supplementary Material
