# Supplementary material for: Hepatic arterial chemotherapy infusion combined with tyrosine kinase inhibitors and PD-1 inhibitors for advanced hepatocellular carcinoma with high risk: a propensity score matching study
Source: Int J Surg. 2024 Jul 12;111(1):104–12. doi: 10.1097/JS9.0000000000001940 (PMC11745606; doi:10.1097/JS9.0000000000001940)
Supplement: Supplementary file 2 [file js9-111-0104-s002.docx]

**Supplementary** **materials**

**Supplementary Table 1.** Univariate and Multivariate Cox regression analyses of the prognostic factors for overall survival and progression-free survival before propensity score matching.

| Variable | OS | | | | PFS | | | |
| --- | --- | --- | --- | --- | --- | --- | --- | --- |
|  | Univariate cox regression | | Multivariate cox regression | | Univariate cox regression | | Multivariate cox regression | |
|  | HR (95% CI) | P value | HR (95% CI) | P value | HR (95% CI) | P value | HR (95% CI) | P value |
| AFP, ng/ml (＜=400 versus ＞400) | 0.94 (0.72-1.23) | 0.642 |  |  | 1.11 (0.88-1.39) | 0.391 |  |  |
| Age, year (<=50 versus >50) | 0.91 (0.7-1.19) | 0.5 |  |  | 0.90 (0.72-1.12) | 0.351 |  |  |
| ALBI (1 versus 2) | 1.24 (0.96-1.61) | 0.105 |  |  | 1 (0.8-1.25) | 0.994 |  |  |
| Child-Pugh class (A versus B) | 1.74 (1.19-2.56) | 0.005 | 1.51 (1.03-2.23) | 0.036 | 1.19 (0.83-1.69) | 0.349 |  |  |
| Distant Metastasis (absent versus present) | 1.20 (0.93-1.56) | 0.167 |  |  | 1.38 (1.11-1.73) | 0.004 | 1.35 (1.08-1.68) | 0.009 |
| Hepatitis B infection (absent versus present) | 0.99 (0.62-1.59) | 0.972 |  |  | 1.08 (0.73-1.61) | 0.688 |  |  |
| Sex (female versus male) | 0.79 (0.58-1.09) | 0.149 |  |  | 0.96 (0.74-1.26) | 0.79 |  |  |
| Tumor number (＜=3 versus ＞3) | 1.61 (1.22-2.13) | 0.001 | 1.66 (1.26-2.19) | <0.001 | 1.46 (1.16-1.84) | 0.001 | 1.44 (1.14-1.81) | 0.002 |
| Tumor size, cm (＜10 versus ＞=10) | 1.03 (0.72-1.46) | 0.881 |  |  | 1.34 (0.98-1.84) | 0.068 | 1.26 (0.92-1.73) | 0.152 |
| Vp4 (absent versus present) | 1.07 (0.81-1.40) | 0.643 |  |  | 0.85 (0.67-1.07) | 0.163 |  |  |
| Vp4 and tumor size ≥10cm (absent versus present) | 1.15 (0.83-1.59) | 0.399 |  |  | 1.02 (0.77-1.36) | 0.892 |  |  |
| Treatment (dural therapy versus triple therapy) | 0.50 (0.38-0.65) | <0.001 | 0.50 (0.38-0.65) | <0.001 | 0.80 (0.64-0.99) | 0.044 | 0.77 (0.62-0.96) | 0.022 |

Any factors that were statistically significant at P <10% in the univariate analysis were candidates for entry into a multivariable Cox analysis.

HR, hazard radio; CI, confidence interval.

**Supplementary Table 2.** Explicit treatment-related adverse events before propensity score matching.

| adverse event | Grade 3-4 | | | | Grade 1-2 | | | |
| --- | --- | --- | --- | --- | --- | --- | --- | --- |
|  | Dual therapy (N=221) | Triple therapy (N=245) | total (N=466) | p | Dual therapy (N=221) | Triple therapy (N=245) | total (N=466) | p |
| Elevated ALT | 25 (11.3%) | 58 (23.7%) | 83 (17.8%) | <0.001 | 103 (46.6%) | 171 (69.8%) | 274 (58.8%) | <0.001 |
| Elevated AST | 32 (14.5%) | 60 (24.5%) | 92 (19.7%) | 0.009 | 119 (53.8%) | 177 (72.2%) | 296 (63.5%) | <0.001 |
| Fever | 5 (2.3%) | 5 (2.0%) | 10 (2.1%) | 1 | 5 (2.3%) | 55 (22.4%) | 60 (12.9%) | <0.001 |
| Nause | 2 (0.9%) | 1 (0.4%) | 3 (0.6%) | 0.929 | 38 (17.2%) | 74 (30.2%) | 112 (24.0%) | 0.002 |
| Vomit | 1 (0.5%) | 17 (6.9%) | 18 (3.9%) | <0.001 | 6 (2.7%) | 66 (26.9%) | 72 (15.5%) | <0.001 |
| Abdominal pain | 8 (3.6%) | 71 (29.0%) | 79 (17.0%) | <0.001 | 19 (8.6%) | 148 (60.4%) | 167 (35.8%) | <0.001 |
| Hyperbilirubinemia | 8 (3.6%) | 15 (6.1%) | 23 (4.9%) | 0.302 | 68 (30.8%) | 139 (56.7%) | 207 (44.4%) | <0.001 |
| Anemia | 6 (2.7%) | 8 (3.3%) | 14 (3.0%) | 0.94 | 43 (19.5%) | 167 (68.2%) | 210 (45.1%) | <0.001 |
| Neutropenia | 19 (8.6%) | 78 (31.8%) | 97 (20.8%) | <0.001 | 38 (17.2%) | 127 (51.8%) | 165 (35.4%) | <0.001 |
| Thrombocytopenia | 31 (14.0%) | 64 (26.1%) | 95 (20.4%) | 0.002 | 54 (24.4%) | 96 (39.2%) | 150 (32.2%) | <0.001 |
| Bleeding | 19 (8.6%) | 4 (1.6%) | 23 (4.9%) | 0.001 | 4 (1.8%) | 27 (11.0%) | 31 (6.7%) | <0.001 |
| Diarrhea | 4 (1.8%) | 16 (6.5%) | 20 (4.3%) | 0.023 | 61 (27.6%) | 78 (31.8%) | 139 (29.8%) | 0.37 |
| Hoarseness | 2 (0.9%) | 0 (0%) | 2 (0.4%) | 0.434 | 10 (4.5%) | 10 (4.1%) | 20 (4.3%) | 0.995 |
| Rash | 1 (0.5%) | 19 (7.8%) | 20 (4.3%) | <0.001 | 37 (16.7%) | 80 (32.7%) | 117 (25.1%) | <0.001 |
| HFS | 22 (10.0%) | 34 (13.9%) | 56 (12.0%) | 0.247 | 78 (35.3%) | 74 (30.2%) | 152 (32.6%) | 0.284 |
| Hypertension | 90 (40.7%) | 31 (12.7%) | 121 (26.0%) | <0.001 | 73 (33.0%) | 83 (33.9%) | 156 (33.5%) | 0.924 |
| RCCEP | 9 (4.1%) | 4 (1.6%) | 13 (2.8%) | 0.188 | 60 (27.1%) | 68 (27.8%) | 128 (27.5%) | 0.966 |
| Hypothyroidism | 14 (6.3%) | 0 (0%) | 14 (3.0%) | <0.001 | 15 (6.8%) | 22 (9.0%) | 37 (7.9%) | 0.482 |
| Fatigue | 0 (0%) | 3 (1.2%) | 3 (0.6%) | 0.284 | 51 (23.1%) | 83 (33.9%) | 134 (28.8%) | 0.014 |
| Hepatitis | 2 (0.9%) | 1 (0.4%) | 3 (0.6%) | 0.929 | 1 (0.5%) | 2 (0.8%) | 3 (0.6%) | 1 |
| Pneumonia | 2 (0.9%) | 1 (0.4%) | 3 (0.6%) | 0.929 | 3 (1.4%) | 0 (0%) | 3 (0.6%) | 0.211 |
| Proteinuria | 15 (6.8%) | 14 (5.7%) | 29 (6.2%) | 0.774 | 88 (39.8%) | 132 (53.9%) | 220 (47.2%) | 0.003 |

P values were calculated using a two-sided Chi-square test.

ALT, alanine aminotransferase; AST, aspartate aminotransferase; HFS, hand-foot syndrome; RCCEP, reactive cutaneous capillary endothelial proliferation.

**Supplementary Table 3.** Explicit treatment-related adverse events after propensity score matching.

| adverse event | Grade 3-4 | | | | Grade 1-2 | | | |
| --- | --- | --- | --- | --- | --- | --- | --- | --- |
|  | Dual therapy (N=194) | Triple therapy (N=194) | total (N=388) | p | Dual therapy (N=194) | Triple therapy (N=194) | total (N=388) | p |
| Elevated ALT | 21 (10.8%) | 43 (22.2%) | 64 (16.5%) | 0.004 | 92 (47.4%) | 129 (66.5%) | 221 (57.0%) | <0.001 |
| Elevated AST | 27 (13.9%) | 45 (23.2%) | 72 (18.6%) | 0.026 | 104 (53.6%) | 138 (71.1%) | 242 (62.4%) | <0.001 |
| Fever | 5 (2.6%) | 4 (2.1%) | 9 (2.3%) | 1 | 5 (2.6%) | 40 (20.6%) | 45 (11.6%) | <0.001 |
| Nause | 2 (1%) | 1 (0.5%) | 3 (0.8%) | 1 | 31 (16.0%) | 64 (33.0%) | 95 (24.5%) | <0.001 |
| Vomit | 1 (0.5%) | 12 (6.2%) | 13 (3.4%) | 0.005 | 5 (2.6%) | 50 (25.8%) | 55 (14.2%) | <0.001 |
| Abdominal pain | 7 (3.6%) | 55 (28.4%) | 62 (16.0%) | <0.001 | 17 (8.8%) | 118 (60.8%) | 135 (34.8%) | <0.001 |
| Hyperbilirubinemia | 8 (4.1%) | 12 (6.2%) | 20 (5.2%) | 0.491 | 59 (30.4%) | 106 (54.6%) | 165 (42.5%) | <0.001 |
| Anemia | 3 (1.5%) | 5 (2.6%) | 8 (2.1%) | 0.721 | 38 (19.6%) | 131 (67.5%) | 169 (43.6%) | <0.001 |
| Neutropenia | 14 (7.2%) | 59 (30.4%) | 73 (18.8%) | <0.001 | 35 (18.0%) | 99 (51.0%) | 134 (34.5%) | <0.001 |
| Thrombocytopenia | 29 (14.9%) | 49 (25.3%) | 78 (20.1%) | 0.016 | 48 (24.7%) | 72 (37.1%) | 120 (30.9%) | 0.012 |
| Bleeding | 15 (7.7%) | 3 (1.5%) | 18 (4.6%) | 0.008 | 4 (2.1%) | 23 (11.9%) | 27 (7.0%) | <0.001 |
| Diarrhea | 4 (2.1%) | 13 (6.7%) | 17 (4.4%) | 0.047 | 53 (27.3%) | 58 (29.9%) | 111 (28.6%) | 0.653 |
| Hoarseness | 2 (1.0%) | 0 (0%) | 2 (0.5%) | 0.478 | 6 (3.1%) | 10 (5.2%) | 16 (4.1%) | 0.444 |
| Rash | 1 (0.5%) | 14 (7.2%) | 15 (3.9%) | 0.002 | 30 (15.5%) | 61 (31.4%) | 91 (23.5%) | <0.001 |
| HFS | 21 (10.8%) | 23 (11.9%) | 44 (11.3%) | 0.873 | 68 (35.1%) | 60 (30.9%) | 128 (33%) | 0.45 |
| Hypertension | 77 (39.7%) | 30 (15.5%) | 107 (27.6%) | <0.001 | 63 (32.5%) | 68 (35.1%) | 131 (33.8%) | 0.668 |
| RCCEP | 8 (4.1%) | 3 (1.5%) | 11 (2.8%) | 0.221 | 52 (26.8%) | 52 (26.8%) | 104 (26.8%) | 1 |
| Hypothyroidism | 13 (6.7%) | 0 (0%) | 13 (3.4%) | <0.001 | 14 (7.2%) | 19 (9.8%) | 33 (8.5%) | 0.467 |
| Fatigue | 0 (0%) | 1 (0.5%) | 1 (0.3%) | 1 | 47 (24.2%) | 61 (31.4%) | 108 (27.8%) | 0.141 |
| Hepatitis | 0 (0%) | 1 (0.5%) | 1 (0.3%) | 1 | 1 (0.5%) | 2 (1%) | 3 (0.8%) | 1 |
| Pneumonia | 2 (1.0%) | 1 (0.5%) | 3 (0.8%) | 1 | 2 (1%) | 0 (0%) | 2 (0.5%) | 0.478 |
| Proteinuria | 11 (5.7%) | 11 (5.7%) | 22 (5.7%) | 1 | 78 (40.2%) | 98 (50.5%) | 176 (45.4%) | 0.053 |

P values were calculated using a two-sided Chi-square test.

ALT, alanine aminotransferase; AST, aspartate aminotransferase; HFS, hand-foot syndrome; RCCEP, reactive cutaneous capillary endothelial proliferation.

**Supplementary Table 4.** Baseline characteristics grouped by tumor response in the triple-therapy group.

| Characteristic | | Triple-therapy group | | | |
| --- | --- | --- | --- | --- | --- |
|  |  | Non-response (N=113) | Response (N=132) | total (N=245) | p |
| Age (Years) | <=50 | 62 (54.9%) | 71 (53.8%) | 133 (54.3%) | 0.968 |
|  | >50 | 51 (45.1%) | 61 (46.2%) | 112 (45.7%) |  |
| Sex | Female | 12 (10.6%) | 13 (9.8%) | 25 (10.2%) | 1 |
|  | Male | 101 (89.4%) | 119 (90.2%) | 220 (89.8%) |  |
| Hepatitis B infection | No | 10 (8.8%) | 8 (6.1%) | 18 (7.3%) | 0.556 |
|  | Yes | 103 (91.2%) | 124 (93.9%) | 227 (92.7%) |  |
| ALBI grade | 1 | 44 (38.9%) | 71 (53.8%) | 115 (46.9%) | 0.028 |
|  | 2 | 69 (61.1%) | 61 (46.2%) | 130 (53.1%) |  |
| AFP (ng/ml) | <=400 | 37 (32.7%) | 47 (35.6%) | 84 (34.3%) | 0.737 |
|  | >400 | 76 (67.3%) | 85 (64.4%) | 161 (65.7%) |  |
| Child-Pugh class | A | 100 (88.5%) | 122 (92.4%) | 222 (90.6%) | 0.406 |
|  | B | 13 (11.5%) | 10 (7.6%) | 23 (9.4%) |  |
| Tumor size, cm | Mean ± SD | 13.2 ± 3.5 | 12.1 ± 3.6 | 12.6 ± 3.6 | 0.012 |
|  | <10 | 14 (12.4%) | 25 (18.9%) | 39 (15.9%) | 0.222 |
|  | >=10 | 99 (87.6%) | 107 (81.1%) | 206 (84.1%) |  |
| Tumor number | <=3 | 32 (28.3%) | 53 (40.2%) | 85 (34.7%) | 0.071 |
|  | >3 | 81 (71.7%) | 79 (59.8%) | 160 (65.3%) |  |
| Vp4 | No | 75 (66.4%) | 79 (59.8%) | 154 (62.9%) | 0.357 |
|  | Yes | 38 (33.6%) | 53 (40.2%) | 91 (37.1%) |  |
| Vp4 and tumor size ≥10cm | No | 89 (78.8%) | 104 (78.8%) | 193 (78.8%) | 1 |
|  | Yes | 24 (21.2%) | 28 (21.2%) | 52 (21.2%) |  |
| Distant Metastasis | No | 40 (35.4%) | 71 (53.8%) | 111 (45.3%) | 0.006 |
|  | Yes | 73 (64.6%) | 61 (46.2%) | 134 (54.7%) |  |
| HAIC cycle | Median (IQR) | 4.0 (2.0 to 6.0) | 6.0 (4.5 to 6.0) | 5.0 (3.0 to 6.0) | <0.001 |

P values were calculated using two-sided Chi-square test, two-sided independent *t*-test and Wilcoxon rank-sum test.

Non-response: stable disease (SD) and progressive disease (PD); Response: complete response (CR) and partial response (PR).

IQR, interquartile range.

HAIC, hepatic arterial infusion chemotherapy.

**Supplementary Table 5.** Univariate and Multivariate Cox regression analyses of the prognostic factors for overall survival and progression-free survival in the triple-therapy group.

| Variable | OS | | | | PFS | | | |
| --- | --- | --- | --- | --- | --- | --- | --- | --- |
|  | Univariate cox regression | | Multivariate cox regression | | Univariate cox regression | | Multivariate cox regression | |
|  | HR (95% CI) | P value | HR (95% CI) | P value | HR (95% CI) | P value | HR (95% CI) | P value |
| AFP, ng/ml (＜=400 versus ＞400) | 1.2 (0.79-1.81) | 0.402 |  |  | 1.35 (0.97-1.88) | 0.075 | 1.29 (0.92-1.82) | 0.14 |
| Age, year (<=50 versus >50) | 0.67 (0.45-1) | 0.048 | 0.58 (0.38-0.87) | 0.008 | 0.71 (0.52-0.97) | 0.03 | 0.78 (0.57-1.08) | 0.131 |
| ALBI (1 versus 2) | 1.84 (1.24-2.75) | 0.003 | 1.67 (1.1-2.54) | 0.016 | 1.1 (0.81-1.5) | 0.533 |  |  |
| Child-Pugh class (A versus B) | 2.02 (1.1-3.71) | 0.023 | 2.08 (1.07-4.03) | 0.03 | 1.22 (0.72-2.08) | 0.46 |  |  |
| Distant Metastasis (absent versus present) | 1.28 (0.86-1.89) | 0.223 |  |  | 1.44 (1.05-1.97) | 0.023 | 1.28 (0.93-1.76) | 0.125 |
| Hepatitis B infection (absent versus present) | 1.37 (0.56-3.36) | 0.494 |  |  | 1.27 (0.67-2.41) | 0.466 |  |  |
| Sex (female versus male) | 1.33 (0.69-2.55) | 0.395 |  |  | 0.88 (0.55-1.41) | 0.595 |  |  |
| Tumor number (＜=3 versus ＞3) | 2.03 (1.31-3.16) | 0.002 | 1.97 (1.26-3.09) | 0.003 | 1.65 (1.18-2.3) | 0.004 | 1.47 (1.05-2.06) | 0.027 |
| Tumor size, cm (＜10 versus ＞=10) | 0.77 (0.47-1.26) | 0.297 |  |  | 1.42 (0.92-2.19) | 0.112 |  |  |
| Vp4 (absent versus present) | 1.41 (0.96-2.09) | 0.082 | 1.13 (0.75-1.69) | 0.56 | 0.82 (0.6-1.14) | 0.235 |  |  |
| Vp4 and tumor size ≥10cm (absent versus present) | 1.3 (0.83-2.04) | 0.254 |  |  | 1.03 (0.7-1.5) | 0.895 |  |  |
| HAIC cycle | 0.76 (0.68-0.84) | <0.001 | 0.75 (0.66-0.84) | <0.001 | 0.86 (0.79-0.94) | 0.001 | 0.87 (0.8-0.95) | 0.003 |

Any factors that were statistically significant at P <10% in the univariate analysis were candidates for entry into a multivariable Cox analysis.

HAIC, hepatic arterial infusion chemotherapy. HR, hazard radio; CI, confidence interval.

**Supplementary Figure 1.**

**
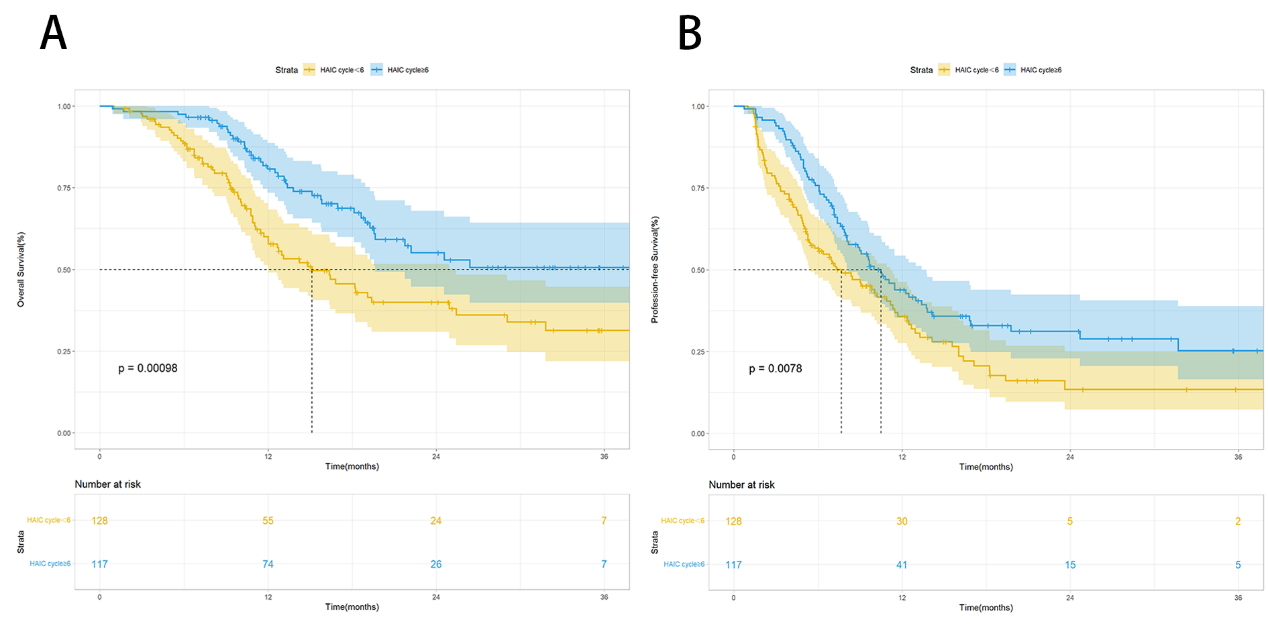
**

**Figure legends:** Kaplan–Meier survival curves comparing OS (A) and PFS (B) among patients underwent higher and lower HAIC cycles in the triple-therapy group.

HAIC, hepatic arterial infusion chemotherapy. OS, overall survival. PFS, progression-free survival.

**Annotation:** The 1-and 2-year OS rates in the low HAIC cycle group were 57.9% and 40.0%, and in the high HAIC cycle were 80.7% and 55.1%, respectively (Supplementary Figure 1A). The 1-and 2-year PFS rates in the low HAIC cycle group were 35.7% and 13.4%, and in the high HAIC cycle were 43.8% and 31.2%, respectively (Supplementary Figure 1B).
